# Effects of copper/graphene oxide core-shell nanoparticles on *Rhipicephalus* ticks and their detoxification enzymes

**DOI:** 10.1038/s41598-025-86560-4

**Published:** 2025-01-27

**Authors:** Haytham Senbill, Amr Gangan, Ahmed M. Saeed, Mohammed E. Gad, Jehan Zeb, Alaa Fahmy

**Affiliations:** 1 Depaertment of Applied Entomology and Zoology, Faculty of Agriculture, Alexandria University, Alexandria, 21545 Egypt; 2 Department of Chemistry, Faculty of Science, Al-Azhar University, Cairo, 11884 Egypt; 3 Department of Zoology and Entomology, Faculty of Science, Al-Azhar University, Cairo, 11884 Egypt; 4 Department of Zoology, Higher Education Department, Government Ghazi Umara Khan Degree College Samar bagh, Lower Dir, Khyber Pakhtunkhwa 25000 Pakistan; 5 Petrochemicals Department, Faculty of Engineering, Pharos University in Alexandria, Alexandria, Egypt; 6 Bundesanstalt für Materialforschung und -prüfung (BAM), Unter den Eichen 87, 12205 Berlin, Germany

**Keywords:** Copper/graphene oxide, Nanopesticides, *Rhipicephalus rutilus*, *Rhipicephalus turanicus*, Acetylcholinesterase, Antioxidants, Electrochemistry, Materials chemistry, Physical chemistry, Surface chemistry, Entomology

## Abstract

Nanopesticides have been recently introduced as novel pesticides to overcome the drawbacks of using traditional synthetic pesticides. The present study evaluated the acaricidal activity of Copper/Graphene oxide core-shell nanoparticles against two tick species, *Rhipicephalus rutilus* and *Rhipicephalus turanicus*. The Copper/Graphene oxide core-shell nanoparticles were synthetized through the solution plasma (SP) method under different conditions. The nanoparticles synthesized at 180 W and 45 min were highly toxic to *Rh. rutilus* and *Rh. turanicus*, with 50% lethal concentration (LC_50_) values of 248.1 and 195.7 mg ml^−1^, respectively, followed by those which were synthesized at 120 W/30 mins (LC_50_ = 581.5 and 526.5 mg ml^−1^), 120 W/15 mins (LC_50_ = 606.9 and 686.7 mg ml^−1^), and 100/45 mins (LC_50_ = 792.9 and 710.7 mg ml^−1^), after 24 h of application. The enzyme assays revealed that 180 W/45 min treatment significantly inhibited the activity of acetylcholinesterase (115 ± 0.81 and 123 ± 0.33 U/ mg protein/min) and superoxide dismutase (290 ± 0.18 and 310 ± 0.92 U/ mg protein/min) in *Rh. rutilus* and *Rh. turanicus*, respectively, as compared with the negative control. The results also revealed a significantly increased catalase activity (895 ± 0.37 and 870 ± 0.31 U/ mg protein/min) in *Rh. rutilus* and *Rh. turanicus*, respectively. The above results indicated that Copper/Graphene oxide core-shell nanoparticles could be a promising alternatives for the management of ticks.

## Introduction

Ticks (Acari: Ixodida) are blood-feeding arthropods with worldwide distribution, infesting virtually all terrestrial vertebrates^1^. They constitute a serious threat to the world economy by causing tremendous economic losses to animals and humans^2^. Direct damages due to tick infestation are represented by blood loss, anemia, inflammation, hypersensitivity, irritation, and skin wounds^3^. Indirectly, they are involved in the transmission of a wide range of pathogenic bacteria, protozoa, viruses, and filarial nematodes^2^. Out of four tick families, Argasidae (soft ticks) and Ixodidae (hard ticks) are considered as economically important^3^.

Ixodid ticks are currently 762 recognized and valid species according to the latest checklists^4^. Members of the genus *Rhipicephalus*comprise 11.2% of the total Ixodidae family^4^. A lack and inaccurate original description of *Rh. sanguineus*led to a global debate about the taxonomic status of at least 17 similar species^5^, including *Rh. rutilus* and *Rh. turanicus*. Recently, the southeastern Europe lineage of *Rh. sanguineus* s.l. was identified to be *Rh. rutilus*^6^and is well-distributed in Africa, including Lower Egypt and the Nile Delta^7^. In Egypt, *Rh. rutilus*, which infests dogs and sheep has been found to be positive for several bacterial and protozoan pathogens^8^ Likewise, *Rh. turanicus* ticks are involved in the transmission of *Hepatozoon* and *Babesia*infections to animal hosts^9,10^.

Numerous control strategies, including chemical acaricides, are usually used to control ticks. However, the unwise usage of these chemicals has enabled ticks to acquire different levels of resistance over the years^11^. Moreover, environmental pollution, and cross-contamination of meat and milk of the treated hosts are of high concern^12^. In response to the problems associated with the unwise use of synthetic acaricide, researchers have been proposing alternative products that could be of great help to control ectoparasites without negativeeffectson their host and the environment^13^. In recent years, nanoparticles have been offered as novel pesticides for pest management^14,15^. They are optimized in terms of extended and sustained release of their active ingredients, while reducing application rates, and often minimizing environmental pollution that might occur with the application of synthetic pesticides^16–18^. Earlier studies confirmed the high efficacy of metal and carbon nanoparticles against arthropods, including biting lice^19^, mosquitoes^20^, beetles^21^, blowflies^22^, and mites^23^. Interestingly, graphene oxide nanoparticles were synergistically used with acaricides, including pyridaben, chlorpyrifos, and β-cyfluthrin to increase their acaricidal efficacy against spider mites^24^. Likewise, graphene nanocarriers of insecticides such as lambada-cyhalothrin and cyfluthrin were found to increase the toxicity of the chemical insecticides against *Helicoverpa armigera*worms by easing the insertion of the active ingredients through the insect cuticle^25^. On the other hand, copper nanomaterials have been shown to be effectiveas multi-action pesticides against *Spodoptera frugiperda*^26^, *Tribolium castaneum*^27^, *Phyllocoptruta oleivora*, *Eutetranychus orientalis*, and *Brevipalpus obovatus*^28^. Recent studies have concentrated on carbon materials like carbon fibers, carbon nanotubes, and graphene due to their potential to enhance the strength and conductivity of copper matrix composites^29–31^. Among these, graphene stands out as a particularly effective reinforcement due to its two-dimensional hexagonal structure, which is made up of sp² hybridized carbon atoms, providing exceptional mechanical strength and electrical conductivity^30,32,33^. This study focused on the acaricidal activity of newly synthesized Copper/Graphene oxide nanoparticles (Cu/GO NPs) against *Rh. rutilus* and *Rh. turanicus* ticks and their consequential effects on key enzymes of the ticks.

## Materials and methods

Dimethylformamide (DMF, 99.5%), acetone (99.5%) and ethanol (absolute) were purchased from El-gomhouria, Cairo, Egypt. Dimethylsulfoxide (≥ 99.9% ), 5,5ʹ-dithiobis-(2-nitrobenzoic acid) (DTNB), acetylthiocholine iodide (ATChI), glucose-6-phosphate dehydrogenase (G-6PD), p-nitroanisole, and sodium dodecyl sulfate were purchased from Sigma–Aldrich, Steinheim, Germany, while HCl, Triton X-100, and sodium phosphate buffer (0.1 M, pH 7.6) from Thermo Fisher Scientific, Massachusetts, USA.

### Tick collection and identification

Adult males and females of *Rh. rutilus* and *Rh. turanicus* ticks were collected using tweezers from naturally infested dogs of Alexandria Governorate, Egypt (31° 11’ 57.05’’ N; 29° 53’ 42.62’’ E) with no history of acaricidal application. The collected unfed adult ticks were morphologically identified with the help of Motic SFC-11 (Motic Asia, Kowloon, Hong Kong) at 40X magnification, following the keys of Filippova^34^ and Walker et al.^9,35^. The ticks were fed on domestic rabbits (*Oryctolagus cuniculus*) to produce the next-generation progeny used in the bioassay.

### Synthesis and characterization of copper / graphene oxide nanoparticles

**Fig. 1:**
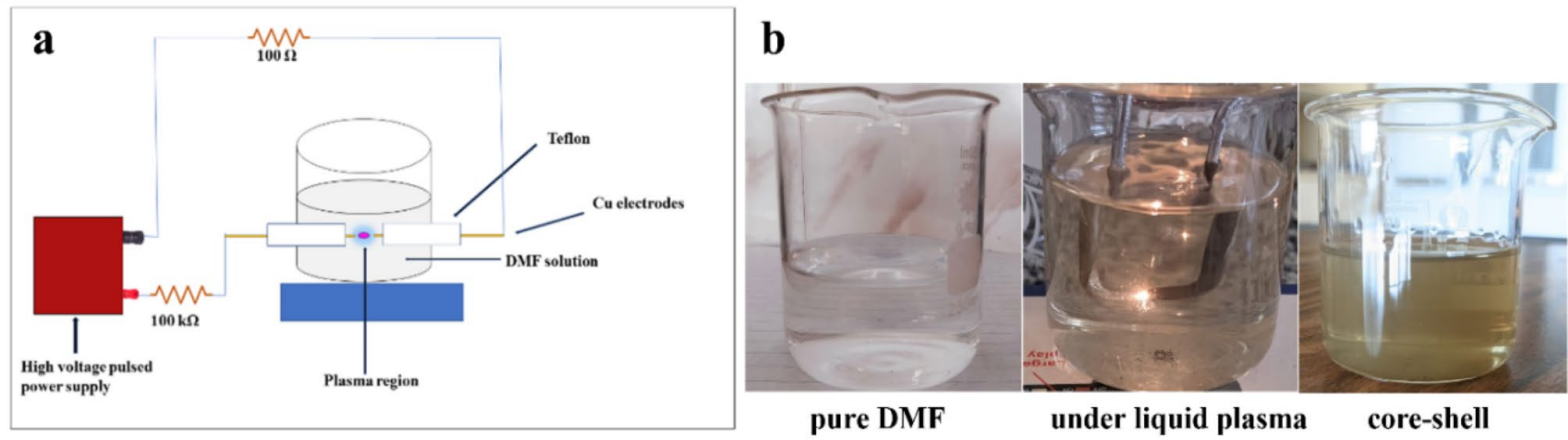
Schematic diagram of solution plasma process (**a**), and Cu/NFG formation process by solution plasma method (**b**).

Copper-coated with NFG was synthesized through the solution plasma (SP) method using two copper wires as an opposite electrode. In the SP process, the pair of electrodes were insulated by Teflon tubes and plugged into a glass reactor. The tips of the electrodes were placed at the center of the reactor with a gap distance of 1.0 mm, as shown in Fig. 1a. The plasma discharge was generated and maintained between the two electrodes which were immersed in 100 mL of DMF by using a homemade DC pulsed power supply. The plasma operating parameters were fixed at a pulse width of 1.0 µs and a repetition frequency of 50 kHz. During the plasma discharge, black solid particles were continuously separated as presented in Fig. 1b. After 15 min of the reaction, the black solid particles were collected and washed with acetone and dried at 60 °C for 12 h.

### Characterization of the synthesized nanoparticles

The chemical structure of the prepared Copper/Graphene oxide (Cu/GO) samples was investigated utilizing the FTIR spectrometer Alpha 2 (Bruker) and the attenuated total reflectance (ATR) mode was employed in the range from 400 to 4000 cm^−1^. The bonding state of all elements on the surface of the samples was characterized by X-ray photoelectron spectroscopy (XPS). The measurements were carried out using a spectrometer purchased from ThermoFisher Scientific, USA, working with Al Kα radiation, with an energy of 1487 eV. The emission voltage and power of this source are set to 11 kV and 220 W, respectively. The pressure was fixed in the analyzing chamber at 10^−7^ Pa throughout the analysis. The fitting of the spectra was carried out employing Casa XPS. The light absorption and optical properties of the specimens were recorded and analyzed through a UV–Visible spectrometer (UV–Vis, Jenway Model 6700). Additionally, the morphologies and the elemental analysis of the prepared samples were investigated using high-resolution transmission electron microscopy (HR-TEM, which connected to Energy-dispersive X-ray (EDX)- JEM-200 Plus LaB6 S) at an accelerating voltage of 200 kV.

### Concentration preparation

Three grams of the formulated nanoparticles were dissolved in 30 ml of 70% Dimethylsulfoxide (DMSO) as a stock solution. Concentration ranges of 50, 200, 500, 700, and 900 mg mL^−1^ were prepared from each stock to study the effect of the nanoparticle materials on the ticks. Abamectin, a synthetic acaricide (Syngenta Agro Co., Basal, Switzerland) diluted in distilled water with the same concentration range was used as a positive control.

### Adult immersion test (AIT)

This experimental work and the animals used in the study (obtained from the farm of Faculty of Agriculture, Alexandria University, Egypt) were performed according to the Alexandria University Research Ethics Review Committee. The ethical approval for this study was granted by the ethical committee on animal care and use at the Faculty of Agriculture, Alexandria University, Egypt (Alex.Agri.1124004306).

The AIT experiments were conducted following Drummond et al.^36^. Briefly, three replicates, each of ten individual adult ticks (unfed; five males: five females for each replicate) were immersed for ten min in 20 mL of each concentration of the nanoparticles material (100/45, 120/15, 120/30, and 180/45). A negative and positive control group of ticks were immersed in 70% DMSO (70% of DMSO was not found toxic to the ticks during the preliminary study) and abamectin, respectively. The treated ticks were then allowed to dry on filter papers, placed in separate petri plates, and incubated at 26°C; 80% RH; 12 h of light:12 h of dark. Mortality was recorded at 24-, 48-, 72-, and 96-h post-treatment. The mortality of ticks was observed based on the total absence of movement, particularly the tick legs.

### Enzymatic activity determinations

The ticks were subjected to enzymatic activity at 48 h post-treatment. Thirty whole ticks (ten/replicate) from each treatment were dealt with as a pool and processed for homogenization DMSO (70%) and abamectin-treated ticks were employed as negative and positive controls, respectively. The homogenate tissues were prepared based on the methods suggested by Anazawa et al.^37^ through crushing with liquid nitrogen, suspended in cold 50 mM Tris-HCl (pH 8.0) containing 0.1% Triton X-100, and centrifuged to remove the precipitate. The activity of acetylcholinesterase (AChE) was determined using a modified Ellman’s method^38^ and modified by Gorun et al.^39^. Briefly, the AChE activity was measured by adding 0.3 mM of 5,5ʹ-dithiobis-(2-nitrobenzoic acid) (DTNB, ) and 0.3 mM of acetylthiocholine iodide (ATChI, ) at 30 °C using a spectrophotometer to measure absorbance at 412 nm over 5 min period.

The monooxygenase activity was assayed to determine the CP450 activity according to the method of Hansen and Hodgson^40^. The standard incubation mixture contained 1 mL sodium phosphate buffer (0.1 M, pH 7.6), 1.5 mL enzyme solution, 0.2 mL NADPH (1 mM final concentration), 0.2 mL glucose-6-phosphate (G.6.P, final concentration, 1 mM) and 50 µg glucose-6-phosphate dehydrogenase (G-6PD). The reaction in the previous mixture was initiated by the addition of p-nitroanisole in 10 µL acetone to give a final concentration of 0.8mM and was incubated for 30 min at 37°C. The incubation period was terminated by the addition of 1 mL HCl (1 N). P-nitrophenol was extracted with CHCl_3_ and 0.5 N NaOH and the absorbance of the NaOH solution was measured at 405 nm. An extinction coefficient of 14.28 mM^−1^cm^−1^ was used to calculate 4-nitrophenol concentration. The enzyme activity was expressed as n mole substrate oxidized /min/mg protein.

The activity of carboxylesterase was determined using the method proposed by Zhang et al.^41^. In summary, a 25 µL solution of carboxylesterase protein (with a final concentration of 2.0 µg/µL protein in 0.1 M PBS buffer, pH 7.5) and 90 µL of 3 × 10^–4^ M substrate solution (either α-naphthyl acetate (α-NA) or β-naphthyl acetate (β-NA) dissolved in 0.1 M PBS buffer) were added to a 96-well microplate and incubated at 30 °C for 30 min. The reaction was stopped by adding 45 µL of freshly prepared diazo blue-sodium lauryl sulfate solution (containing 2 parts of 1% fast blue B salt and 5 parts of 5% sodium dodecyl sulfate solution). After 15 min of incubation at room temperature, the absorbance value of the hydrolysis product α-NA or β-NA was measured at 600 nm–550 nm, respectively. The superoxide dismutase (SOD) activity was assayed in the tissue homogenates as units per milligram protein (U/mg protein/ min) by the method suggested by Nishikimi et al.^42^. The catalase (CAT) activity was assayed in the tissue homogenates as units per milligram protein (U/mg protein/min) using the method described by Aebi^43^, and the glutathione peroxidase (GPX) activity was evaluated in the tissue homogenates using the method suggested by Paglia and Valentine^44^. Results were defined as units per milligram protein (U/mg protein/min).

### Statistical analysis

Probit analysis according to Finney^45^ was used to estimate the lethal concentration (LC_50_) values using MedCalc Statistical Software ver. 19.2.6 (MedCalc Software Ltd, Ostend, Belgium). Data of the enzymatic activities were analyzed using analysis of variance (ANOVA), followed by Tukey post-hoc test. The significant levels were set at *P* ≤ 0.05.

## Results and discussion

### Characterization of Cu/GO nanoparticles

During the plasma discharge in DMF using a pair of Cu electrodes, black particles were continuously generated. According to the literature, the SP process could produce nitrogen-doped carbon materials from the nitrogen-containing organic precursors^46^. The black particles could be suggested to be the combination of nitrogen-doped carbon and Cu nanoparticles, which could occur through the decomposition and recombination of DMF and the simultaneous sputtering of Cu electrodes together^47^.

#### Fourier-transform infrared spectroscopy (FT-IR)

**Fig. 2:**
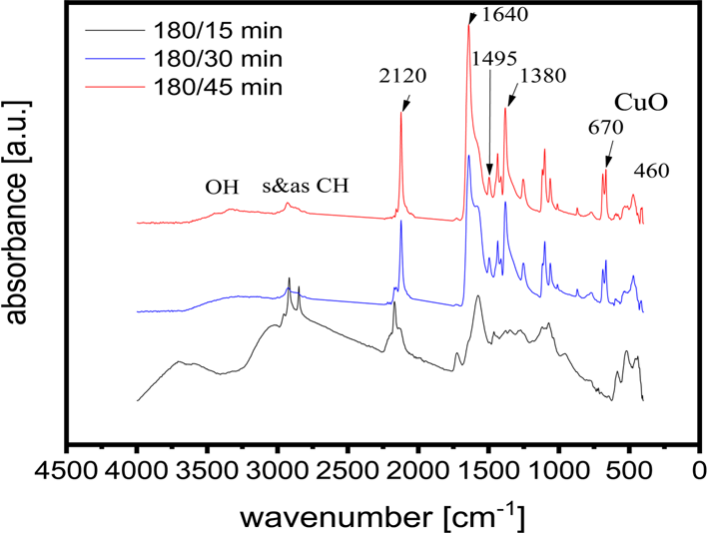
FT-IR spectra of the Cu/GO at different times of plasma discharge and (50 kHz, and 180 W).

The changes in the FTIR spectral features (Fig. 2) at different plasma discharge times (15, 30, and 45 min) can provide insights into the evolution of the chemical composition and the degree of functionalization of the Cu/NFG composite during the plasma treatment process. The results showed an absorbance of new peaks after 30 min at 460 and 670 cm^−1^ due to the Cu–O bond in the monoclinic crystal structure of CuO suggesting the successful synthesis of CuO core. The absorption peaks at 1380 and 1495 cm^−1^were attributed to C–N in the N-doped (functionalized) graphene (NFG). The increased intensity of the peak at 1640 corresponds to the C = N stretching vibration, suggesting the existence of NFG^48^. Furthermore, the broad peak around 3332 cm^−1^ and small peak around 2922 cm^−1^ correspond to OH and CH stretching, respectively in the graphene. The intensity of all observed peaks is increased after 45 min of plasma operating time, indicating that it is the best preparation time for Cu-CuO-NFG catalyst (Fig. 2).

#### X-ray photoelectron spectroscopy (XPS)

**Fig. 3:**
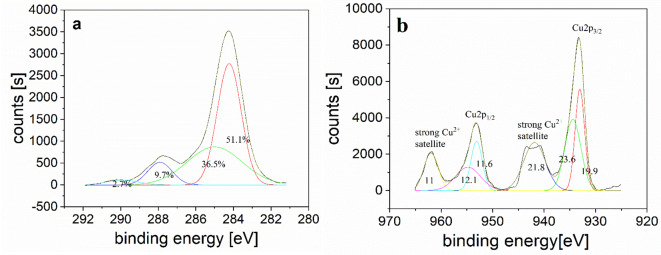
High resolution XPS spectra of the C1s peak for Cu/-NFG (**a**) and Cu2p peak for Cu/GO (**b**), with relative area under the peaks (45 min, 50 kHz and 180 W).

The surface chemical compositions and elemental bonding states of the obtained samples were demonstrated by XPS analysis. The XPS results showed the nitrogen and oxygen contents in the sample (Cu/GO at 180 W, 50 kHz, and 45 min), suggesting that Cu-NFG could be produced by the decomposition and recombination of the molecules of DMF during the SP process (Table 1). Furthermore, deconvolution was applied to the high-resolution XPS spectra to understand the atomic-level details and detecting the chemical states of the elements. The C1s spectra were deconvoluted at least into four peaks as shown in Fig. 3a. The strong peak at 284.2 eV is assigned to C–C of the carbon sp2 structures of NFG (Fig. 3a)^49^. In addition, the occurrence of C–N, C = O, and O–C = O bonding peaks at 285, 286.6, and 289.8 eV, respectively, was detected, indicating the functionalization of the graphene by nitrogen and oxygen (carbons attached to nitrogen and different oxygen-containing moieties)^50,51^. The high-resolution Cu 2p3/2 spectra which were deconvoluted into two peaks at 933.06 eV and 953.1 eV which assigned to Cu 2p3/2 and Cu 2p1/2, respectively (Fig. 3b). The presence of CuO is confirmed by appearance of two strong satellite peaks at 941.9 eV, and 961.9 eV, respectively^52^. In addition, Cu(0) may also observed at 933.06 eV (19.9%) and Cu(II) at 934.4 eV (23.6%) and 941.9 eV (21.8%), respectively^53^ indicating that oxidized Cu core (CuO) exists in Cu-NFG which was in good agreement with FT-IR results.

**Table 1:** Surface composition Cu/GO at 180 W, 50 kHz, and 45 min, as determined by XPS

Name	Peak BE	FWHM eV	Area (*P*) CPS.eV	Atomic %
Cu2p3	934,19	3,99	261397,2	11,68
O1s	531,23	4,18	77721,04	14,28
N1s	399,35	3,54	21333,5	5,9
C1s	285,35	3,83	66247,36	31,08
Al2p3	76,69	4,37	31123,32	37,05

#### UV–visible spectrometry (UV–Vis.)

**Fig. 4:**
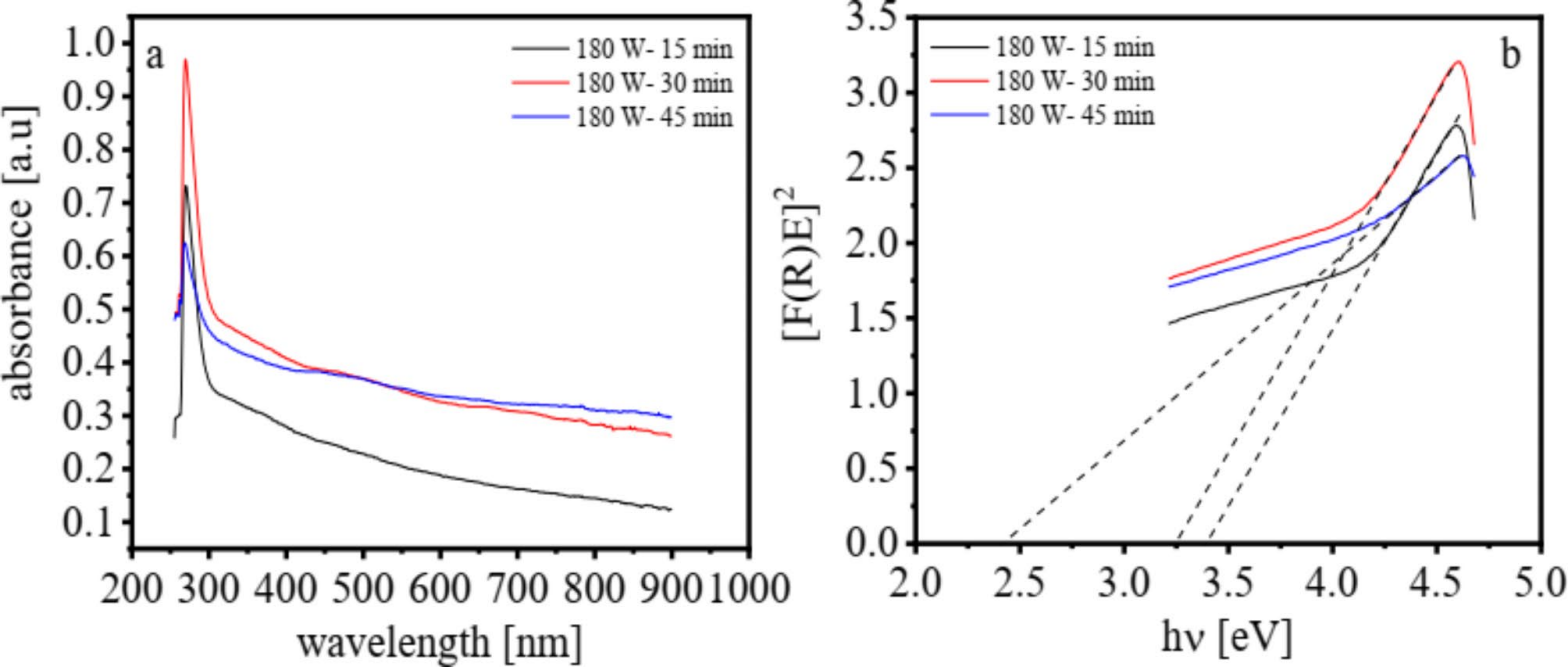
UV–Vis. absorption spectra (**a**), and the band gap (**b**) of Cu/GO nanoparticles at 180 W pulsed plasma discharge, frequency = 50 kHz and different time (15, 30 and 45 min).

The optical properties were elucidated by measuring UV–Vis. absorption spectra of graphene (G) and graphene oxide (GO) in addition to Cu nanoparticles and the results were presented in Fig. 4a. According to literatures, the GO sheets demonstrate an absorption peak centered at 273 nm and a shoulder at about 325 nm, which could be assigned to the π → π* transitions of aromatic C–C bonds and the n → π* transitions of C = O bonds, respectively. Furthermore, there is a broad shoulder in the range from 425 to 550 nm which might correspond to the Cu nanoparticles with different sizes (Fig. 4a). Furthermore, it was observed that the band gap value is reduced with increasing the time of plasma discharge (3.4, 3.2, and 2.4 eV for 15, 30, and 45 min) (Fig. 4b) indicating that the activity of the nanomaterials as an electrocatalyst was increased as the plasma discharge time was increased^54–56^.

#### Transmission electron microscopy (TEM) and energy-dispersive X-ray spectroscopy (EDX)

**Fig. 5:**
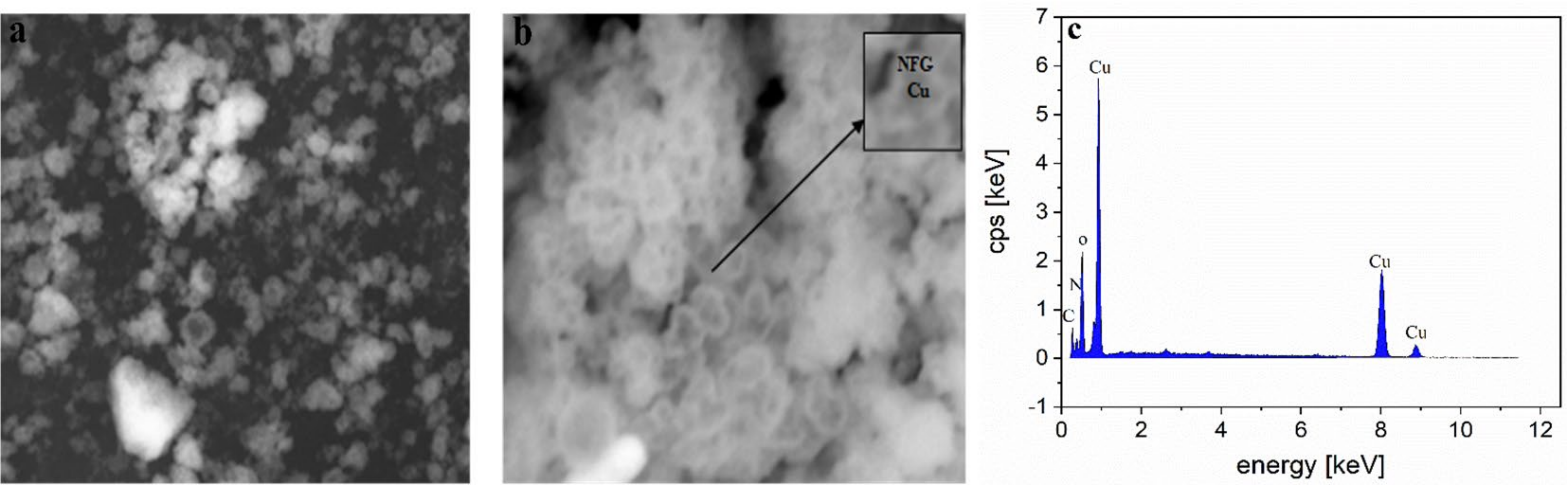
TEM images at low (**a**) and high (**b**) resolution, and EDX analysis of the Cu-NFG nanoparticles (**c**) (45 min, 50 kHz and 180 W).

The TEM showed the morphology of Cu-NFG. The black Cu–core and a grayish carbon–shell appeared indicating the successful formation of the core shell (Fig. 5a, b). The average particle size of Cu-NFG was measured to be 29 nm. The results of the EDX analysis revealed that the Cu was covered by NFG as indicated by the presence of N as compared to pristine Cu^57^ (Fig. 5c).

A potential route for the SP process to generate a core-shell nanostructure might be suggested based on the findings of the Cu-NFG characterization. Two primary processes could combine to produce Cu encapsulated by NFG. The first was carbonization, which created a shell of carbon materials, and the second was plasma sputtering, which produced metal atoms at the tip of metal electrodes and formed the metal-core. The DMF molecules broke down in the plasma zone (higher temperature) during the SP process. Radicals and fragments, which are crucial species for the synthesis and growth of carbon materials, were produced when the bonds between the carbon atoms in DMF were broken^58^. Later, those fragments were propagated and recombined to form a carbon framework. Nevertheless, its dispersion to the surrounding solution phase of low temperature (room temperature), prevented the carbon structure from developing and extending. Furthermore, the important species that resulted from the breakdown of DMF^59^ are CN radicals, a crucial part of nitrogen doping in carbon materials, which are formed by the insertion of nitrogen atoms during the initial growth stage and the generation of graphitic planes, which ultimately result in the formation of nitrogen doped carbon particles.

Without the need for additional chemicals, such as reducing agents, Cu nanoparticles could be synthesized by plasma sputtering at the electrode tips to form them as the core component of Cu-NFG. During the plasma discharge, the energetic atoms, ions, and electrons in the plasma gas phase hit, battered, and knocked off the metal atoms at electrodes^60^. The nucleation and subsequent crystal development to a bigger cluster and nanoparticle may be caused by the ejected metal atoms. At the same time, the formation of graphene was aided by the growth of metal crystals. According to XPS, TEM, and EDX analyses, the metal’s surface serves as a substrate for the adsorption of a pre-formed carbon layer (Figs. 3 and 5)^61^. As shown by FT-IR and UV-Vis analysis, it was found that longer plasma discharge times resulted in more carbonization and sputtering, which in turn led to more combination and formation of Cu-NFG (Figs. 2 and 4).

### Toxicity of Cu/GO NPs to *Rh. rutilus* and *Rh. turanicus* ticks

The toxicological assay of the synthesized nanoparticles against *Rh. rutilus* and *Rh. turanicus* are shown in Table 2.

**Table 2:** Toxicity of Cu/GO NPs against *Rhipicephalus rutilus* and *Rh. turanicus* adults after 24-, 48-, 72-, and 96 h post-treatment.

Treatment		LC_50_ mg ml^−1^ (95% CL)		LC_95_ mg ml^−1^ (95% CL)		Slope ± SE		X^2^		*P* value (*P*> 0.05)	
Synthesis conditions	Time interval (hours)	*Rh. rutilus*	*Rh. turanicus*	*Rh. rutilus*	*Rh. turanicus*	*Rh. rutilus*	*Rh. turanicus*	*Rh. rutilus*	*Rh. turanicus*	***Rh. rutilus***	*Rh. turanicus*
100/45	24	792.9 (533.2-1,550)	710.7 (518.2-1,825)	13,547 (9,421 − 18,550)	12,863 (8,014–17,185)	2.38 ± 2.12	1.56 ± 0.85	7.870	5.536	0.0821	0.0282
	48	707.7 (506.2-1,388)	624.3 (422.8-1,478)	12,557 (8,175 − 19,713)	12,086 (7,417 − 20,691)	3.38 ± 2.12	1.52 ± 0.79	6.868	5.581	0.0843	0.0232
	72	631.0 (418.7-1,593)	529.9 (406.5-1,125)	12,151 (8,628 − 21,952)	11,789 (7,102 − 19,731)	1.39 ± 0.78	0.70 ± 0.51	4.790	2.128	0.0298	0.0466
	96	619.2 (355.9-1,382)	482.9 (364.3-1,674)	11,884 (7,514 − 18,195)	11,439 (8,319 − 21,827)	1.63 ± 0.83	0.85 ± 0.51	6.419	3.151	0.0222	0.0277
120/15	24	606.9 (327.7–972.6)	686.7 (411.3-1,919)	12,404 (7,252 − 18,435)	11,247 (8,556 − 22,753)	0.43 ± 0.75	0.45 ± 0.42	1.040	1.173	0.1318	0.1401
	48	561.8 (402.9-1,444)	628.3 (316.8-1,024)	11,248 (9,522 − 21,82)	10,902 (7,614 − 18,731)	0.45 ± 0.42	0.62 ± 0.42	1.173	2.216	0.1401	0.0790
	72	406.1 (226.8-1,031)	571.8 (316.2–981.5)	10,935 (8,332 − 19,282)	10,388 (7,226 − 17,332)	0.34 ± 0.40	0.26 ± 0.40	0.662	1.420	0.2543	0.3263
	96	376.5 (208.8–861.6)	401.2 (200.5–981.8)	10,322 (6,224 − 17,312)	9,752 (5,405 − 16,242)	0.40 ± 0.40	0.45 ± 0.39	1.025	1.283	0.2364	0.2111
120/30	24	581.5 (382.3–888.4)	526.5 (316.2-1.052)	10,049 (7,127 − 18,379)	9,605 (6,913 − 15,922)	0.62 ± 0.42	0.33 ± 0.40	2.216	0.662	0.0790	0.2543
	48	512.0 (286.1–825.5)	487.6 (229.8–912.8)	9,751 (5,722 − 14,823)	9,398 (4,341 − 13,725)	0.46 ± 0.40	0.56 ± 0.40	1.137	2.000	0.1736	0.1415
	72	436.8 (195.8–909.4)	316.8 (185.9–732.2)	9,002 (4,923 − 16,253)	8,336 (3,941 − 17,323)	0.51 ± 0.40	0.30 ± 0.39	1.675	0.605	0.1603	0.4456
	96	387.6 (195.6–835.7)	197.7 (86.5–722.6)	8,854 (4,392 − 13,823)	7,521 (3,221 − 12,825)	0.56 ± 0.44	0.50 ± 0.39	2.000	1.634	0.1415	0.2505
180/45	24	248.1 (93.7–629.3)	195.7 (79.5–533.1)	5,111 (2,812-8,322)	4,667 (1,923-7,271)	0.98 ± 0.41	0.76 ± 0.40	5.881	3.661	0.0259	0.0871
	48	142.3 (88.9–577.6)	87.8 (59.5–377.2)	4,886 (925.3-6,822)	3,697 (869.3-5,001)	0.76 ± 0.56	0.57 ± 0.39	3.725	2.055	0.1036	0.2709
	72	91.2 (50.5–381.2)	75.7 (44.8–288.7)	4,002 (992.3-6,100)	2,188 (810.7-4,021)	0.69 ± 0.52	0.91 ± 0.42	3.040	4.955	0.1763	0.0959
	96	59.1 (35.8–273.8)	47.5 (29.7–255.7)	3,514 (901.5-5,152)	1,956 (682.7-3,720)	0.96 ± 0.42	0.94 ± 0.43	5.310	4.832	0.1024	0.1365
Abamectin	24	33.1 (20.2–50.8)	25.3 (17.8–30.8)	110.5 (77.9–243.8)	96.7 (75.1–195.8)	0.83 ± 0.31	1.01 ± 0.14	0.521	0.662	0.0514	0.2145
	48	20.9 (11.8–30.7)	18.6 (10.5–21.8)	98.2 (79.8–192.3)	73.0 (62.8–110.1)	1.77 ± 0.15	1.35 ± 0.16	0.669	0.715	0.1579	0.8820
	72	13.7 (9.8–19.2)	11.0 (8.5–17.9)	90.5 (73.3–119.2)	88.2 (62.5–100.9)	1.81 ± 0.11	1.57 ± 0.40	0.488	0.522	0.0335	0.3157
	96	10.1 (6.2–15.8)	8.8 (3.3–16.8)	71.9 (58.1–92.3)	70.5 (55.9–94.8)	1.97 ± 0.02	2.01 ± 0.19	0.371	0.261	0.1176	0.2525

LC_50_: 50% Lethal concentration; CL: Confidence limits; SE: Standard error; *X*^*2*^: Chi-square, *P*: Significance probability values (*P* < 0.05).

The NPs synthesized at 180 W/45 mins had the lowest LC_50_ values against *Rh. rutilus* (248.1 mg ml^−1^) and *Rh. turanicus* (195.7 mg ml^−1^), which indicates a higher acaricidal activity, followed by those which were synthesized at 120 W/30 mins (LC_50_ = 581.5 and 526.5 mg ml^−1^), 120 W/15 mins (LC_50_ = 606.9 and 686.7 mg ml^−1^), and 100/45 mins (LC_50_ = 792.9 and 710.7 mg ml^−1^), respectively, after 24 h of application. However, the efficacy of the NP (180 W/ 45 min) was lower than the positive control (abamectin), which achieved LC_50_ values of 33.1 and 25.3 mg ml^−1^ against *Rh. rutilus* and *Rh. turanicus*, respectively, after 24 h of application. Furthermore, the results indicated time-dependent effects of the NPs against *Rh. rutilus* and *Rh. turanicus*, with the most toxic effect occurring at 96 h of application based on the LC_50_ values (Table 2). Overall, the acaricidal results demonstrated that, the activity of Cu/GO NPs against *Rh. rutilus* and *Rh. turanicus* may depend on the synthesized conditions and duration of exposure. Cu and GO NPs distinct physical and chemical properties, as well as, their methods of penetrating may be responsible for their observed acaricidal activity against *Rh. rutilus* and *Rh. turanicus*. Nanoparticles are usually within the range of 1 to 100 nm in size. They are chemically active and toxic because of their minuscule size and increased reactivity due to their large surface area to volume ratio^62–64^. Besides, the high surface area of graphene (up to 2630 m²/g)^65^enables it to be in close association with biological membranes, which enhances its efficiency as a pesticide formulation system^66^. Furthermore, the shape of the nanoparticles equally affects their mode of action and dispersion. For instance, Cu NPs that are spherical might have an easier time passing through cellular anatomical boundaries than those which are irregularly shaped or too large^67,68^. Moreover, Gr NPs may be functionalized and have carboxyl and hydroxyl groups, which can enhance their ability to be chemically reactive and interact with biological systems^69,70^.

A potential synergistic interaction between Cu and GO may also be responsible for their acaricidal activity against *Rh. rutilus* and *Rh. turanicus*. Indeed, synergistic and/or enhanced effects have been found with graphene oxide in combination with Cu and other compounds. For instance, an enhanced antibacterial effect of GO–Cu NPs composites was found against *Pseudomonas syringae*^71^. It has also been demonstrated that the combination of graphene oxide and synthetic acaricides, including pyridaben, chlorpyrifos, and β-cyfluthrin produced synergistic effects, enhancing the acaricidal activity of the acaricides against spider mites^71^. The ability of graphene to serve as a carrier for other compounds comes from its tailorable surface chemistry and biocompatibility in terms of its 2D structures that are supported by atomic thickness and notable high surface area (2600 m^2^/g)^72^, which not only plays as a carrier for other active agents, but also considered as a compound of pesticidal properties^71^. A previous study has proposed that the synergistic effects of graphene oxide could be a result of the disruption of the cement layers of the arthropod cuticle, leading to quick water loss and improving the penetration of the toxic compounds to the pest body^71^.

When acting alone, graphene has been reported to have insecticidal activity against *Rhyzopertha dominica*, *Sitophilus oryzae* and *Tr. castaneum*, causing 100% mortality to these stored products insects after 21 days of exposure to 500 and 1000 ppm treatments^73^. Besides, Cu NP was found to have a toxic effect against several hematophagous pests, including *Anopheles subpictus* (LC_50_ = 0.95 mg/L), *Culex quinquefasciatus* (LC_50_ = 1.01 mg/L) and *Rhipicephalus microplus* (LC_50_= 1.06 mg/L)^74^. CuO NP was also found to have acaricidal activity against *Rh. microplus* (LC_50_ = 4.30 mg/L), *Haemaphysalis bispinosa* (LC_50_ = 9.50 mg/L) and *Hippobosca maculata* (LC_50_= 11.13 mg/L)^75^.

### Effects of Cu/GO NPs on the ticks enzymes

The full profile of the enzymatic activity of *Rh. rutilus* and *Rh. turanicus* treated with Cu/GO NPs is provided in Table 3. The results showed that the NPs synthesized at 180 W/45 min can either inhibit or enhance the enzymatic activity of both tick species. For instance, 180 W/45 min treatment significantly inhibited the activity of AChE (115 ± 0.81 and 123 ± 0.33 U/ mg protein/min) and SOD (290 ± 0.18 and 310 ± 0.92 U/ mg protein/min) in *Rh. rutilus* and *Rh. turanicus*, respectively, as compared with the negative control. The results also revealed a significantly increased activity of CAT (895 ± 0.37 and 870 ± 0.31 U/ mg protein/min) in *Rh. rutilus* and *Rh. turanicus*, respectively, compared to the negative control. Although not statistically significant from the negative control, inhibitory effects were recorded for carboxylesterase, monooxygenase, and GPX after exposure to Cu/GO NPs (180 W/45 min) (Table 3).

**Table 3:** Effects of different synthesized Cu/GO nanoparticles on the enzymes of *Rhipicephalus rutilus* and *Rh. turanicus* ticks.

Enzyme	Treatments ± SE											
	Control (Negative)		100 W/45 mins		120 W/15 mins		120 W/30 mins		180 W/45 mins		Abamectin	
	*Rh. rutilus*	*Rh. turanicus*	*Rh. rutilus*	*Rh. turanicus*	*Rh. rutilus*	*Rh. turanicus*	*Rh. rutilus*	*Rh. turanicus*	*Rh. rutilus*	*Rh. turanicus*	*Rh. rutilus*	*Rh. turanicus*
AChE (U/mg protein/min)	240 ± 0.25^a^	245 ± 0.11^a^	170 ± 0.92^a^	173 ± 0.81^a^	164 ± 0.73^a^	153 ± 0.71^a^	131 ± 0.28^a^	140 ± 0.64^a^	115 ± 0.81^b^	123 ± 0.33^b^	65 ± 0.16^c^	59 ± 0.39^c^
Carboxylesterase (U/mg protein/min)	305 ± 0.15^a^	300 ± 0.18^a^	398 ± 0.17^a^	400 ± 0.55^a^	405 ± 0.63^a^	411 ± 0.41^a^	462 ± 0.33^a^	475 ± 0.33^a^	243 ± 0.39^a^	237 ± 0.08^a^	95 ± 0.24^b^	81 ± 0.73^b^
Monooxygenase (n mole substrate oxidized/min/mg protein/min)	120 ± 0.11^a^	124 ± 0.25^a^	205 ± 0.57^a^	215 ± 0.27^a^	225 ± 0.12^a^	230 ± 0.23^a^	237 ± 0.74^a^	242 ± 0.17^a^	100 ± 0.29^a^	91 ± 0.73^a^	55 ± 0.36^b^	46 ± 0.15^b^
Catalase (U/mg protein/min)	450 ± 0.38^a^	437 ± 0.44^a^	450 ± 0.73^a^	490 ± 0.71^a^	541 ± 0.27^a^	574 ± 0.37^a^	749 ± 0.62^a^	751 ± 0.79^a^	895 ± 0.37^b^	870 ± 0.31^b^	101 ± 0.22^c^	92 ± 0.82^c^
Glutathione peroxidae (U/mg protein/min)	150 ± 0.66^a^	147 ± 0.72^a^	151 ± 0.48^a^	145 ± 0.48^a^	147 ± 0.73^a^	162 ± 0.12^a^	175 ± 0.57^a^	181 ± 0.46^a^	115 ± 0.30^a^	120 ± 0.88^a^	67 ± 0.71^b^	55 ± 0.71^b^
Superoxide dismutase (U/mg protein/min)	705 ± 0.17^a^	695 ± 0.81^a^	460 ± 0.81^a^	472 ± 0.10^a^	405 ± 0.18^a^	390 ± 0.77^a^	366 ± 0.11^a^	351 ± 0.61^a^	290 ± 0.18^b^	310 ± 0.92^b^	124 ± 0.19^c^	119 ± 0.91^c^

SE: Standard error; Different letters indicate statistically significant differences between rows (*P* < 0.05).

Although the mechanisms by which NPs exert their toxic effects have not yet been fully elucidated, it is believed that this may occur through the inhibition of AChE^76^. AChE is responsible for the degradation of the neurotransmitter acetylcholine at the neural synapses in the central nervous system of arthropods^77^. It is an important target for some groups of insecticides/acaricides, such as organophosphates (Ops) and carbamates^77^. The inhibition of AChE by toxic compounds can lead to acetylcholine accumulation, disruption of neurotransmission, and hyperstimulation of nicotinic receptors^78^. Therefore, the inhibition of AChE by our synthesized Cu/GO (180 W/45)NPs may affect several neural/neuromuscular, and physiological processes, leading to the functional breakdown of the nervous system and death of the ticks^79^. It has been reported that the inhibitory effects caused by NPs are primarily due to their ability to adsorb or interact with AChE^76^. This adsorption or interaction is probably due to their high affinity for the enzyme^76^. Nevertheless, further studies are needed to elucidate the relationship between the inhibitory effect and the acaricidal mechanisms of the synthesized Cu/GO NPs.

In addition to neurotoxicity, oxidative stress has been regarded as one of the mechanisms for NPs toxicity^80^. Nanoparticles can induce oxidative stress by generating elevated levels of reactive oxygen species (ROS)^80^. SOD and CAT are important antioxidant enzymes. SOD protects against oxidative stress, by converting superoxide anions (O_2_^•−^) to hydrogen peroxide (H_2_O_2_) and oxygen^81,82^. The reduced SOD activity of *Rh. rutilus* and *Rh. turanicus *induced by Cu/GO (180 W/45) treatment may lead to the accumulation of superoxide radicals^83^, as these radicals are toxic to the cells and can lead to cell death^84^. On the other hand, CAT can converts H_2_O_2_, which is another form of ROS that can induce oxidative stress into water and oxygen^85^. The increase in the production of this enzyme after the treatment in this study may be an attempt to eliminate the potentially harmful superoxide and H_2_O_2,_ indicating an antioxidant response by the ticks.

The varied enzymatic activity of the NPs may be because of their different sizes and morphology, and their ability to penetrate different organelles of the ticks.

## Conclusions

Nanoparticles have been offered as alternative methods in controlling different pests, including ticks, as it has lesser or no environmental stress compared to traditional synthetic chemicals. The results of the present study demonstrate that Cu/GO NPs, especially the NPs synthesized at 180 W/45 mins exhibited acaricidal activity against *Rh. rutilus* and *Rh. turanicus*, with the potential of inhibiting AChE and SOD activity. Therefore, it could be considered as a suitable compound for effective management of ticks. However, further studies are needed to evaluate the toxic effects of CU/GO NPs on other pests and non-target organisms, as well as, in real field conditions.

## Data Availability

All data generated or analyzed during this study are included in this published article.
